# Collagen Injections for Rotator Cuff Diseases: A Systematic Review

**DOI:** 10.3390/clinpract15020028

**Published:** 2025-01-28

**Authors:** Rocco Aicale, Eugenio Savarese, Rosita Mottola, Bruno Corrado, Felice Sirico, Raffaello Pellegrino, Danilo Donati, Roberto Tedeschi, Luca Ruosi, Domiziano Tarantino

**Affiliations:** 1 Department of Orthopaedic and Trauma Surgery, Casa di Cura di Bernardini, 74121 Taranto, Italy; eugenio.savarese@gmail.com; 2 Public Health Department, University of Naples Federico II, 80131 Naples, Italy; rosi.mottola@studenti.unina.it (R.M.); bruno.corrado@unina.it (B.C.); felice.sirico2@unina.it (F.S.); 3 Department of Medicine and Surgery, LUM University, 70010 Casamassima, Italy; r.pellegrino@lum.it; 4 Physical Therapy and Rehabilitation Unit, Policlinico di Modena, 41125 Modena, Italy; danilo.donati@unimore.it; 5 Clinical and Experimental Medicine PhD Program, University of Modena and Reggio Emilia, 41125 Modena, Italy; 6 Department of Biomedical and Neuromotor Sciences, Alma Mater Studiorum, University of Bologna, 40126 Bologna, Italy; roberto.tedeschi2@unibo.it; 7 Humanitas Research Hospital Department of Biomedical Sciences, 20090 Milan, Italy; luca.ruosi@humanitas.it; 8 Department of Orthopedic Rehabilitation, Campolongo Hospital, 84025 Marina di Eboli, Italy; drdomizianotarantino@gmail.com

**Keywords:** rotator cuff, rotator cuff tendinopathy, supraspinatus tendinopathy, rotator cuff partial-thickness tear, collagen, collagen injections

## Abstract

Background: Because of its anatomy and function, the rotator cuff (RC) is vulnerable to considerable morbidity. The prevalence of RC diseases (RCDs) among the general population is 5–39%, reaching over 30% in patients older than 60. The aim of the present systematic review is to investigate the effects of the use of collagen injections in the treatment of RCDs. Methods: A systematic search of scientific electronic databases (such as PubMed, Scopus and Web of Science) was performed up to November 2024, following the Preferred Reporting Items for Systematic Reviews and Meta-Analyses (PRISMA) guidelines. Two independent authors conducted the search and assessed the articles. The inter-rater reliability for the quality assessment was measured using Cohen’s kappa coefficient, while the Modified Coleman Methodology Score (CMS) was applied to evaluate the methodological quality of the articles included in this systematic review. Results: A total of eight articles were included, with the overall quality of the included articles being evaluated as fair. Despite the use of different types of collagen and injection protocols, as well as the different scores applied, each included study showed clinically relevant improvements. However, given the high degree of heterogeneity of the included studies, we cannot draw conclusions regarding which type of collagen and injection protocol are best for RCD treatment. Discussion: Collagen administration for RCDs seems to be effective at reducing pain and improving function, as well as the tendon structure, especially in partial tears and RC tendinopathy. High-quality, prospective studies with long-term follow-up are necessary to validate the findings of the articles included in this systematic review.

## 1. Introduction

The rotator cuff (RC) consists of the subscapularis, supraspinatus, and infraspinatus tendons and teres minor muscle, and its function is to dynamically stabilize the glenohumeral joint while avoiding humeral head migration and contributing to shoulder abduction and internal and external rotations [1]. The RC is particularly susceptible to significant morbidity because of its anatomy and function, often resulting from a combination of extrinsic (e.g., anatomical variations, such as acromial morphology, os acromiale, and acromial spurs, which contribute to bony impingement or direct pressure on the surrounding soft tissue) and intrinsic factors (originating within the tendon itself, such as tensile overload, aging, compromised microvascular supply, trauma, or degenerative changes) [2,3].

Rotator cuff diseases (RCDs) are the third most prevalent musculoskeletal pathologies, following lower back pain and knee pain. Their prevalence among the general population ranges from 5% to 39% and increases with age, exceeding 30% among individuals over 60 years old. The majority of cases involve RC tendinopathy (RCTP) or partial- and full-thickness rotator cuff tears (PTRCTs or FTRCTs) [4,5,6,7].

PTRCTs can be divided into bursal, intra-tendinous, and articular tears. They are more common and painful compared to FTRCTs, with a prevalence of 13% to 32% among the adult population [8,9,10]. Several studies have demonstrated that the 80% of PTRCTs progress into FTRCTs despite conservative treatment [11].

The treatment of choice for RCDs is still debated and relies on several factors, including tear degree and size, patient symptoms and needs, and functional loss [12,13].

Arthroscopic cuff repair is the most common and accepted surgical treatment, with proven satisfactory results [14,15], but post-operative re-tears represent an important complication, with an incidence between 34.2% and 40% [16], seriously impacting the rehabilitation process and patients’ quality of life [17,18]. Given the risks related to surgery (such as reduced strength of RC tendons) and the significant risk of recurrence, conservative treatment is usually the first choice, especially for older adults [19,20]. Various studies report good results for the management of both PTRCTs and FTRCTs [4,21], with a surgical option usually being considered when the conservative treatment has not exhibited an effect within the first 5–12 weeks [22,23,24].

Conservative options include pharmacotherapy (such as non-steroidal anti-inflammatory drugs and analgesics), physiotherapy [25,26,27], therapeutic exercise [28,29,30], and injections of various drugs, such as corticosteroids (CS), platelet-rich plasma (PRP), hyaluronic acid (HA), and collagen [31,32,33,34,35].

CS injections provide transient pain relief; however, their use does not modify the natural history of the disease, with recent evidence suggesting a potential role in accelerating the degenerative process in the tendon [36].

PRP alleviates symptoms and slows the degeneration of the tendon better than CS administration and prolotherapy [37,38,39,40]. HA exhibits anti-inflammatory and adhesion-prevention properties [41], and it plays a crucial role in promoting cell differentiation and growth, enhancing type I collagen expression in tendon-derived cells, and supporting tendon and bone healing [42,43,44,45,46]. The use of injectable collagen in the tendon itself or in the subacromial bursa exhibits a positive effect that reduces collagen’s degenerative process [47,48,49].

Several types of collagens (types I, II, III, and V) can be found in fibrous tissues such as tendons, ligaments, and skin. Type I collagen, a major component of the extracellular matrix (ECM) of tendons, directly influences the structural and mechanical properties of tendons [21]. In the RC’s ECM, type I collagen represents more than 95% of the total amount of collagen, whereas the remaining 5% consists of collagen types III and V [50,51].

Atelocollagen, a soluble type I collagen, is the most commonly used injectable collagen because of its excellent biocompatibility, low immunogenicity, prolonged half-life, and high resistance to enzymatic degradation [52,53,54]. Several advantages have been reported with the use of highly purified atelocollagen in terms of collagen–cell interactions and its few adverse effects, such as bruising, redness, swelling, tenderness, or itching, which are, however, very unlikely to occur in patients affected by musculoskeletal disorders, especially RCDs [47,55]. Collagen injections induce regenerative pathways by stimulating tenocyte proliferation and migration, via the synthesis of endogenous collagen, and by the restoration of collagen fibers in damaged tendons [23,36,56,57]. Collagen administration at the target site activates integrin receptors on fibroblast cell membranes [58], triggering a cascade of growth factors that stimulate the synthesis of endogenous collagen [59]. This process ultimately repairs damaged collagen fibers and promotes their proper alignment [60,61,62].

In animal models, the direct administration of atelocollagen at the site of an RC tear improved tendon healing and remodeling, as demonstrated by immunohistochemistry and histopathological analyses [47,63]; a possible explanation of this effect may be a facilitation to re-create tendon continuity at the injured site, decreasing peritendinous adhesions and improving muscle activity.

Collagen injections were also used, with good reported outcomes, for the treatment of large and massive RC tears, with the collagen patches implanted arthroscopically [47,64], as well as for the treatment of tennis elbow [65,66] and plantar fasciitis [59,67].

Evidence of synergic effects between collagen and PRP has been reported, suggesting that PRP can positively influence cellular mitogenic activity, enhance collagen production, and improve the ratio of collagens I/III [68]. Furthermore, injections of combined drugs, such as collagen and PRP or collagen and HA, may promote regeneration of the native insertion site, prevent scar tissue formation, and increase the biomechanical strength [9,69,70,71]. The aim of the present study is to systematically review the use of collagen injections for the treatment of RCDs in humans, given the growing interest and use of infiltrative collagen for treating musculoskeletal disorders in recent years, as well as the need to find a valid, safe, and cost-effective alternative to the currently proposed conservative treatments. We hypothesized that the use of collagen injections allows for the achievement of better clinical and functional outcomes in patients with RCTP, PTRCTs and FTRCTs. To date, this is the first systematic review conducted using PRISMA guidelines regarding the use of collagen injections for RCDs.

## 2. Materials and Methods

Study design

**Figure 1:**
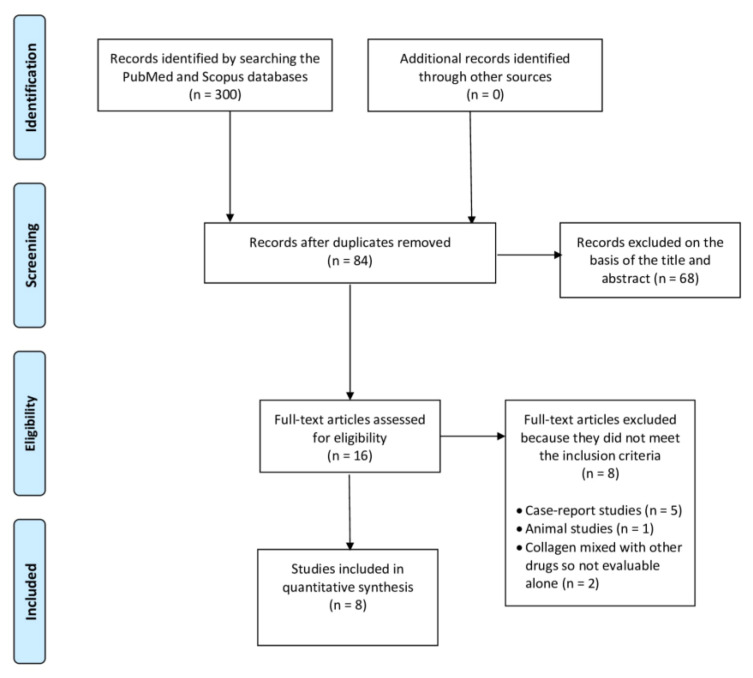
Flowchart of the process for the inclusion of the studies.

This systematic review and its procedures were designed and conducted in accordance with the Preferred Reporting Items for Systematic Reviews and Meta-Analyses (PRISMA) guidelines [72,73,74]. The PRISMA flow diagram is provided in Figure 1, while the PRISMA checklist can be found in the Supplementary Materials [75]. The research protocol has been registered with the International Prospective Register of Systematic Reviews (PROSPERO), under registration number: CRD42023470461.

Eligibility criteria

This review includes randomized clinical trials (RCTs), prospective and case-series studies, with a minimum follow-up of one month. Comparative studies with other injections therapies such as PRP were also included.

Articles such as editorials, technical notes, letters to authors, narrative reviews, systematic reviews, case reports, and animal or cadaveric studies that did not report clinical outcomes of the use of collagen for RCDs were excluded.

Information sources

Potential studies were identified through a search of electronic databases, including the Cochrane Central Register of Controlled Trials (CENTRAL), EMBASE, MEDLINE, PEDro, Web of Science, Scopus, PubMed, and CINAHL. A comprehensive search of all databases was conducted from their inception through to November 2024, with no language restrictions. Additionally, the reference lists of the relevant studies were reviewed for other potential studies, but none were identified.

Search strategy

The strategy had two components including terms for collagen and RCDs. Keywords for population were “Rotator cuff” [MeSH] OR rotator cuff disease*[all fields] OR rotator cuff tendinopathy*[all fields] OR rotator cuff tear*[all fields]. The keywords for the intervention were “collagen” [MeSH] OR “atelocollagen” [MeSH].

Types of participants

This study included patients with a diagnosis of an RCD, defined as RCTP, PTRCTs, or FTRCTs.

Types of interventions

For inclusion, collagen had to be administered to at least one group in the RCTs. Studies in which the effects of collagen alone could not be evaluated (such as a mixture of collagen and another drug compared with collagen alone or another drug) would not be included.

Types of comparison controls

Comparison groups were classified into active and inactive controls according to the “Cochrane Handbook for Systematic Reviews of Interventions” [76]. An inactive control was defined as either no injection or arthroscopic repair without collagen injection. Active control was defined as the use of alternative injection solutions, such as PRP [71] or an acellular dermal matrix allograft [77].

Outcomes measures

The primary outcome was a reduction in pain, measured by the numeric analogue scale (NAS, 0–10) or visual analogue scale (VAS, 0–10). Secondary outcomes included the constant score (CoS) and the American shoulder and elbow surgeons score (ASES), when available. Other scores were evaluated on a case-by-case basis depending on the ones used in the included studies. The outcomes were evaluated at baseline and at final follow-up for each of the included studies.

Study selection and data extraction

Two independent authors (R.A. and D.T.) performed the search and evaluated the articles. Researchers experienced in conducting systematic reviews (E.S., B.C., F.S., and R.P.) resolved any cases of uncertainty. Initially, the investigators reviewed the abstracts of the articles, selecting relevant studies based on the inclusion and exclusion criteria, and then compared their selections with those of other investigators. Two weeks later, the same studies were re-evaluated to confirm agreement. No disagreements were observed among the investigators.

One investigator (R.A.) input data extracted from the full-text articles into structured tables in an Excel spreadsheet for descriptive analysis of each study. The sample sizes, types of management and collagen used, time of follow-up, clinical and functional outcomes before and after treatment, adverse events and complications extracted from the retrieved articles are presented in Table 1. A second investigator (E.S.) independently double-checked the extraction of primary data from all the articles. Doubts and inconsistencies were grouped and solved via another round of revision performed together by the two investigators. All authors participated in drafting the text. All results that were compatible with each outcome domain in each study were sought. A *p*-value < 0.05 was considered statistically significant. The results are presented in Table 1 for a comparison of the progression from baseline to last follow-up. All results are reported for the baseline and last follow-up, highlighting significant differences. The analysis of the level of evidence was conducted according to the Oxford Centre for Evidence-Based Medicine Levels of Evidence [78].

Quality assessment

The Modified Coleman Methodology Score (MCMS) was used to evaluate the quality of the articles included in this PRISMA-based systematic review [81]. The MCMS was used to assess the quality of the articles found in the present study. The assesment of the methodologies used 10 criteria, with a total score between 0 and 100 (which indicates that the study largely avoids chance, various biases, and confounding factors). The final scores were categorized as excellent (85–100 points), good (70–84 points), fair (55–69 points), or poor (<55 points).

The MCMS’ criteria were modified to make them reproducible and relevant for the present systematic review. We changed, for example, the “description of surgical technique” criterion to “description of injection technique”. More details about the MCMS (such as the definitions for each criterion, along with the scoring system) are reported in the Supplementary Materials [82]. Two authors (D.T. and R.A.) independently applied the MCMS, and a final score was reached by consensus. The MCMS is calculated using ten different criteria (study size, follow-up, number of procedures, type of study, diagnostic certainty, description of the injection technique, rehabilitation and compliance, outcome criteria, outcome assessment, and selection process), with a maximum total possible score of 100 [81]. Then, the agreement on the quality assessment between the two reviewers was evaluated using Cohen’s kappa coefficient.

## 3. Results

Eligible studies

After the initial literature search, a total of 300 potentially relevant citations were identified. After removal of duplicate records, 84 articles were identified. Then, following a preliminary check of the titles and abstracts, 68 articles were excluded, since they did not investigate outcomes related to the use of collagen injections for RCDs. Finally, after further screening, another eight articles were excluded because they did not meet the inclusion criteria, with a total of eight articles being included in the present systematic review (Figure 1).

Among the eight excluded studies, five were case-report studies, so their outcomes could not be considered reliable. One article was excluded because it was conducted on animals. Finally, two studies were excluded because they combined collagen with other drugs (such as HA); therefore, the effects of only collagen could not be evaluated.

Quality of the included studies

The inter-rater (R.A. and D.T.) reliability for the quality assessment, evaluated using Cohen’s K coefficient, was optimal (0.9). The raters were blinded to the other reviewer’s ratings.

The results of the MCSMS are reported in Table 2.

There was a large range in the MCMS values, from 52 to 76, with a mean of 63.3 ± 8.9, which is regarded as fair (55–69 points). Some of the selected studies involved relatively small patient cohorts, unclear outcome criteria and assessments, suboptimal patient selection processes, and low-quality evidence. Consequently, a meta-analysis was not conducted.

Characteristics of the included studies

Detailed descriptions of the characteristics of the included studies are provided in Table 1. Of the eight articles retrieved, four studies were retrospective [12,36,48,77], three were prospective studies [49,79,80] reporting the results after a different number of collagen injections (from one to four), while a single study was an RCT [71]. The countries in which the included studies were conducted were South Korea [48,77,79], Italy [36,49,80], and Poland [71].

Four studies evaluated the effects of collagen injections for PTRCTs [48,71,79,80], with one of these being an RCT in which patients were treated using collagen injections alone or combined with PRP or PRP alone [71]. One prospective study [79] evaluated the use of collagen for PTRCTs at different concentrations or without injection therapy, while one retrospective [48] study evaluated collagen injections alone.

Two retrospective studies reported the outcomes of patients treated via arthroscopic rotator cuff repair for FTRCTs and who received either a single collagen injection [12], or a single acellular dermal matrix injection [77], or no injection after surgery.

The effects of the collagen injections on RCTP were investigated in one case-series and one prospective study [36,49]. Buda et al. [49] divided patients on the basis of the simple *shoulder* test (SST), reporting better outcomes in patients treated using two single injections, in approximately two weeks, with the worst SST recorded at baseline.

The follow-ups ranged from a minimum of two months [48] to a maximum of 24 months [79]. The total number of patients enrolled in the included studies was 551, with a minimum of 15 patients [48] and a maximum of 129 patients [77]. The mean age of the patients recruited in the included studies was 57.77 ± 3.93.

Clinical Assessment

The initial assessments of the patients were performed in all of included studies via US or magnetic resonance imaging (MRI) assessments that confirmed the presence of PTRCTs, FTRCTs, or RCTP, and four studies also considered clinical signs and symptoms of RC pathology [36,71,79,80]. Only one study reported the use of specific tests for the clinical assessment of RCDs, such as Neer’s and Jobe’s tests [36].

Injection technique

Six studies used an in-plane, ultrasound (US)-guided injection technique [36,48,49,71,79,80], while in the studies in which collagen injection followed the arthroscopic repair, it was delivered through an arthroscopic visualization [12,77].

**Figure 2:**
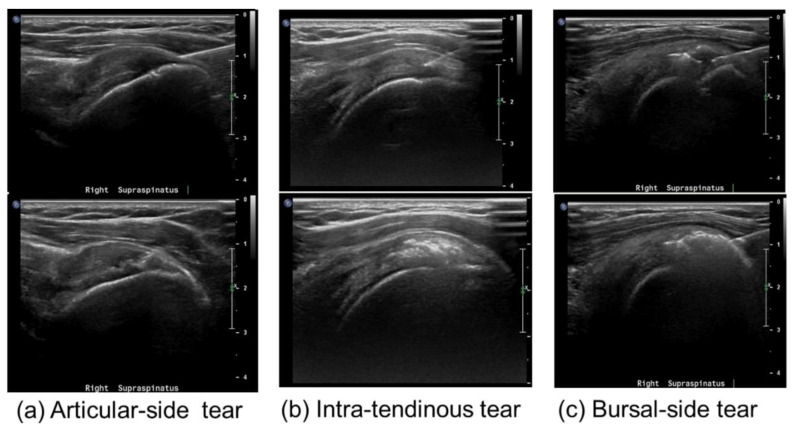
Examples of US-guided collagen injection techniques. Picture retrieved from Chae et al. [48].

In four studies, collagen was injected directly at the tear site [36,48,79,80], in two studies into the subacromial bursa [49,71], and in another two studies at the bone–tendon interface (Figure 2) [12,77].

Comparing the injection techniques, collagen administered into the subacromial bursa for treating PTRCTs [71] and RCTP [49] showed significant improvements in pain and function for both conditions despite the use of various injections protocols and types of collagen administered. Furthermore, no adverse effects related to the injections were observed.

When collagen was injected intra-tendinous for treating PTRCTs [48,79,80] and RCTP [36], improvements in pain and function for both conditions were seen despite different injections protocols and type of collagen administered. However, a higher grade of complications (post-injection pain and progression of the tear size in one patient) compared to subacromial injections was noticed.

In two studies, a single injection was performed at baseline [48,79], in another two, a single injection at baseline was administered after arthroscopic repair [12,77], and in three studies either two [49,80], three [71], or four injections [36] were administered.

Adverse events

A 57% post-injection pain (8/15) score was reported for only a single study [48], while a range from 11.5% [12] to 13.6% [77] for RC re-tears after arthroscopic repair followed by collagen injection was found. Finally, one study [80] reported a progression of the supraspinatus tendon tear with a maximum diameter of >1 cm in a single patient.

Rehabilitation

In one study, the rehabilitation protocol after injection consisted of progressive stretching exercises and posterior capsular stretching, comfortable passive ROM, and strengthening exercises with resistance bands [79], while in another study, pain-free ROM, postural exercises, and scapular stabilization exercise were allowed [71]. In the study in which collagen was injected after the arthroscopic repair [12], the shoulder was immobilized for six weeks using an abduction brace, with early passive ROM permitted within a tolerable range, and active-assisted ROM exercises started after six weeks, while strengthening exercises began after three months.

Primary and secondary outcomes’ evaluation

Various outcomes were assessed, such as range of motion (ROM) [48,79]; VAS; NAS; numeric rating scale (NRS) [12,48,71,79,80]; ASES [48,79]; Korean shoulder score (KSS) [12,48]; CoS and Constant–Murley (CM) scores [36,48]; disabilities of the arm, shoulder, and hand (DASH) and quick-disabilities of the arm, shoulder, and hand (Q-DASH) scores [36,71]; simple shoulder test (SST) [48,49]; shoulder pain and disability index (SPADI) [80]; and the EQ-5D-5L (descriptive part—Utility Index and EQ-VAS 0–100) questionnaire [71].

In five out of the seven studies in which the VAS or NRS were evaluated, the scores decreased at final follow-up compared to the baseline, surpassing the threshold for a minimal clinically important difference (MCID) [83], especially in the short-term [12,36,49,71,80]. Regarding other scores evaluated for shoulder function, such as the DASH, Q-DASH, ASES, CoS, SST, or SPADI, the scores in some studies [36,49,71,79,80] increased significantly from baseline to final follow-up, surpassing the respective MCID [84,85,86,87,88].

In three studies [36,71,79], significant changes in the tendon size and structure were found at the last follow-up using an US or MRI assessment. However, these radiological changes were not observed in the other two studies at the final follow-ups [12,48].

Collagen versus active controls

In the study by Godek et al. [71], collagen injections for PTRCTs were compared to collagen plus PRP and PRP alone. Analysis of the evolution of the NRS among the groups showed a significant reduction in pain intensity, primarily during the first six weeks of follow-up (*p* < 0.001), but no statistically significant differences were observed among the groups (*p* = 0.870). Similarly, the Q-DASH results demonstrated a consistent reduction in the mean values at each measurement point (*p* < 0.001), with no significant differences among the groups (*p* = 0.997). For the EQ-5D-5L VAS subscore, no statistically significant differences were found among the groups at any measurement point. The most notable changes occurred within the six weeks after the last injection (*p* < 0.001).

Although no significant differences were detected in the primary outcomes, there was a trend toward greater improvement in the collagen plus PRP and PRP alone groups between 12 and 24 weeks. Additional outcomes included RC discontinuity (n = 3, one case in each group) and rotator cuff regeneration (n = 22 in the collagen plus PRP group, n = 20 in the collagen group, and n = 23 in the PRP group). The authors conclude that combined therapy with collagen and PRP for PTRCTs is no more effective than monotherapy at reducing pain or improving mobility, self-care, and usual activities.

In the study by Aldhafian et al. [77], patients with FTRCTs were treated by arthroscopic repair only or arthroscopic repair together with collagen or acellular dermal matrix allograft injection. Functional outcomes, including the VAS for pain, ASES score, KSS, and CS score, improved for all three groups compared to preoperative assessments at the final follow-up, with the most consistent improvements observed at the 12-month follow-up. However, no significant differences were found among the groups.

## 4. Discussion

The aim of the present study was to systematically review the use of collagen injections for RCDs, reporting their functional outcomes, alone or in combination with other management modalities. The outcomes of the included studies highlight the paucity of evidence on the effectiveness and safety of collagen injections for RCDs.

Despite variations in collagen types, injection protocols, and assessment scores, all studies included in this systematic review demonstrated that collagen administration effectively reduces pain and improves function in the short to medium term for treating RCDs.

RCDs are among the most prevalent and disabling musculoskeletal disorders, yet their optimal treatment remains a topic of ongoing debate [89,90]. Among conservative treatments, different injection therapies have been proposed over the past few years for the treatment of RCDs, but they have achieved a controversial level of effectiveness according to the scientific literature [91]. Since very few injection therapies have proven effective for RCDs [32,92], collagen injections may represent an effective therapeutic option.

All of the evaluation tools scored differently across the included studies, with statistically significant improvements found for all of scores [36,71,77,79,80] or only for some [12,48].

The US [36,48,71] and MRI [12,79] evaluations were also performed during the follow-up to assess the RC repair’s integrity, with a significant decrease in the tear size reported in three studies [36,71,79].

Five studies [36,49,71,79,80] showed both clinical and radiological improvements for PTRCTs and RCTP with the use of collagen, while non-significant changes were found for only a single article [48] on PTRCTs. When patients treated with collagen injections were compared to non-treated patients, the outcomes were significantly better [77,79]. These outcomes were also observed in two case-reports on the use of injectable collagen for PTRCTs [23,58] in which a complete healing of the tendon tear at the last follow-up, along with improvements in shoulder pain and function, were reported. Therefore, the use of collagen injections for PTRCTs and RCTP appears to be reasonable, especially for patients with worse baselines [49], even if the quality of the studies was relatively low, so several questions still need to be addressed.

Kim et al. [12] compared patients treated with a single collagen injection after RC arthroscopic repair for FTRCTs with patients who underwent arthroscopic repair only, and although patients treated with collagen reported reduced pain at two weeks after surgery, no significant difference in the healing rate of the RC tear at six months post-operatively was found. Surprisingly, a 11.5% re-tear rate was found in the group treated with collagen versus 6.7% in the non-collagen group, even if the difference was not statistically significant.

These outcomes contradict those found by Jeong al. [9], who compared patients treated for FTRCTs with combined collagen and HA after RC arthroscopic repair to patients treated with HA alone or with no injections, reporting no difference in terms of the clinical outcomes at 1-year follow-up. However there was a higher rate of RC tears in the groups treated with HA alone and with no injections, while no re-tears were found in the group treated with combined collagen and HA. Therefore, the authors stated that the co-administration of collagen and HA effectively improved healing of the RC and increased the integrity of the RC repair site. In the same study, at the 3-month follow-up, the authors performed intra-articular CS injection in patients with shoulder pain and ROM limitation; notably, they administered a significantly lower number of CS injections in the group treated with combined collagen and HA. This finding is noteworthy, as emerging evidence suggests that corticosteroids may have tenotoxic effects, including an increased risk of tendon rupture, enhanced tenocyte necrosis, and reduced cell viability [93,94,95].

Despite the good outcomes reported in the included studies in this review, the use of collagen for RCDs remains questionable, since some studies reported conflicting results. For this reason, other types of injections, such as PRP, may be preferable according to two recent systematic review on the efficacy of injections for RC tears [32,92]; however, in one of the included studies [71], patients with a PTRCT were treated with either a combination of collagen and PRP, collagen alone, or PRP alone. No statistically significant differences were observed among the groups for the primary outcomes, with the combined collagen and PRP therapy demonstrating a similar level of effectiveness as monotherapy with either collagen or PRP.

Strengths and limitations

The primary strength of this article is that it is the first systematic review to highlight the potential benefits of collagen injections for RCDs. As no previous reviews on this topic have been published, this work serves as a foundational piece for future research. Furthermore, the extensive scope of this review, encompassing RCTP, as well as PTRCTs and FTRCTs, offers a thorough framework for understanding the clinical and functional improvements following collagen administration for various RCDs. Finally, the comprehensive details on the type of collagen used and the injection protocols adopted in each study included in this review, as reported in Table 1, can help clinicians select the most appropriate treatment option in their clinical practice.

The present review is not without its limitations. First, the quality of the included studies was relatively low, with only one level-I study [71], one level-III [79] study, and six level-IV studies [12,36,48,49,77,80], preventing definitive recommendations regarding the use of collagen injections for RCDs. Furthermore, there was a great heterogeneity in terms of the type of collagen used and the number of injections administered, and even when the same type of collagen was administered, the injection protocol was different [36,49,71]. Even in studies in which the same specific RC issue was addressed [48,71,79], the type of collagen used was different. For this reason, we cannot draw conclusions regarding which collagen and injection protocol is the best choose for the conservative treatment of RCDs.

Finally, the heterogeneity of the studied populations, as well as the absence of a control group, in many of the included studies represent an important limitation.

## 5. Conclusions

Despite a relatively low number of studies and the low quality of the evidence, collagen administration for the conservative treatment of RCDs exhibited positive trends related to reducing pain and improving function during follow-ups, as well as improving tendon structure, with the most satisfactory results seen for PTRCTs and RCTP when patients experienced worse shoulder symptomatology at baseline.

In each study, all of the evaluated scores, or at least some of them, improved significantly with good, reported outcomes, but given the low level of evidence, recommendations regarding the correct indication for the use of collagen for RCDs cannot be defined.

High-quality studies that include long-term follow-ups, such as RCTs, are needed to confirm the outcomes of the included articles. However, since the best conservative treatment for RCD, especially involving injections, remains debated, collagen injections may represent a viable and safe therapeutic option.

## Figures and Tables

**Table 1:** Main results and outcomes of each study.

Study Name	Patient No.	Follow-Up	Groups	Collagen Used	Intervention	Scores at Baseline	Scores at Last Follow-Up	Adverse Events
Kim et al. (2019) [12]	121	VAS: 3 days, 1 and 2 weeks KSS: 3, 12, 24 months	Arthroscopic repair plus collagen injection (Group I, *n* = 61) Arthroscopic repair alone (Group II, *n* = 60) FTRCT	3 mL of 3% porcine type-I atelocollagen	Single injection at baseline (after arthroscopy)	Group I VAS: 5.3 ± 2.1 KSS: 63.0 ± 15.1 Group II VAS: 6.3 ± 1.7 KSS: 61.5 ± 15.2	Group I VAS: 1.2 ± 1.0 KSS: 80.1 ± 9.4 Group II VAS: 3.2 ± 1.7 KSS:82.3 ± 11.2	Group I: 7 re-tears (11.5%) Group II: 4 re-tears (6.7%)
Kim et al. (2020) [79]	94	3, 12, and 24 months	0.5 mL collagen injection (Group I, *n* = 32) 1 mL collagen injection (Group II, *n* = 30) No injection (Group III, *n* = 32) PTRCT	0.5 or 1 mL of 3%, porcine type-I atelocollagen	Single injection at baseline	Group I VAS: 4.1 ASES: 61.9 CoS: 68.1 Group II VAS: 3.6 ASES: 63.5 CoS: 65.8 Group III VAS: 3.4 ASES: 62.9 CoS: 68.4	Group I VAS: 2.1 ± 1.2 ASES: 82.5 ± 12.3 CoS: 89.0 ± 6.9 Group II VAS: 1.4 ± 1.1 ASES: 79.3 ± 8.3 CoS: 82.0 ± 10.1 Group III VAS: 3.3 ± 2.5 ASES: 65.5 ± 8.5 CoS: 62.5 ± 11.5	not reported
Chae et al. (2020) [48]	15	2 months	Collagen injections PTRCT	1 mL atelocollagen + 1 mL of lidocaine	Single injection at baseline	ASES: 57.0 KSS: 64.6 CoS: 56.4 VAS: 4.2 SST: 6.6 FVAS: 6.3	ASES: 60.4 KSS: 68.5 CoS: 58.9 VAS: 3.7 SST: 6.9 FVAS: 7.1	Post-injection pain (57%, 8/15)
Corrado et al. (2020) [36]	18	2 weeks, 1 and 3 months	Collagen injections RCTP	2 mL, porcine type-I atelocollagen	4 injections (one a week for 4 weeks in a row)	CoS: 53.11 ± 12.7 DASH: 37.72 ± 19	CoS: 75 ± 12.9 DASH: 18.67 ± 13	Not reported
Godek et al. (2022) [71]	82	6 weeks, 3 and 6 months	Collagen plus PRP injections (Group I, *n* = 28) Collagen injections (Group II, *n* = 27) PRP injections (Group III, *n* = 27) PTRCT	2 mL, porcine type-I atelocollagen	3 injections (one a week for 3 weeks in a row)	Group I VAS: 74% QuickDASH: 37 NRS: 5 Group II VAS: 68% QuickDASH: 42 NRS: 5.5 Group III VAS: 71% QuickDASH: 41 NRS: 6	Group I VAS: 82% QuickDASH: 15 NRS: 1.5 Group II VAS: 80% QuickDASH: 20 NRS: 2 Group III VAS. 86% QuickDASH: 20 NRS: 1.8	No complications
Aldhafian et al. (2023) [77]	129	3, 6, and 12 months for all groups Last follow-up (months): Group I: 21.6 ± 5.1 Group II: 20 ± 6.3 Group III: 18.3 ± 3.2	Arthroscopic repair only (Group I, *n* = 36) Arthroscopic repair plus collagen injection (Group II, *n* = 44) Arthroscopic repair with acellular dermal matrix allograft injection (Group III, *n* = 49) FTRCT	1 mL atelocollagen	Single injection at baseline (after arthroscopy)	Group I VAS: 4 ASES: 58 CoS: 62 KSS: 61 Group II VAS: 4 ASES: 61 CoS: 68 KSS: 68 Group III VAS: 4 ASES: 62 CoS: 68 KSS: 68	Group I VAS: 2 ASES: 80 CoS: 76 KSS: 75 Group II VAS: 3 ASES: 74 CoS: 79 KSS: 81 Group III VAS: 3 ASES: 76 CoS: 73 KSS: 73	Re-tear rates after 12 months: Group I: 19.4% (7 of 36) Group II: 13.6% (6 of 44) Group III: 20.4% (10 of 49) Adverse events were not detected in any groups.
Buda et al. (2023) [49]	71	1 and 6 months	Collagen injections ➢ Group I (SST < 42, *n* = 23) ➢ Group II (43 < SST < 74, *n* = 28) ➢ Group III (SST > 75, *n* = 20) RCTP	4 mg/2 mL, bovine collagen, low molecular weight (<3 kDa)	2 injections (one at baseline and one between 9 and 17 days after the first one)	Overall population VAS at rest: 4.25 ± 3.10 VAS during movement: 6.56 ± 1.47 VAS at night: 5.33 ± 2.98 CoS: 63.76 ± 12.50 SST: 54.14 ± 20.16 Group I VAS at rest: 6.35 ± 2.29 VAS during movement: 7.26 ± 4.09 VAS at night: 6.56 ± 4.48 CoS: 51.52 ± 59.17 SST: 30.43 ± 40.58 Group II VAS at rest: 4.28 ± 2.07 VAS during movement: 6.57 ± 3.96 VAS at night: 5.03 ± 3.04 CoS: 65.32 ± 74.10 SST: 56.79 ± 72.58 Group III VAS at rest: 1.90 ± 0.95 VAS during movement: 5.85 ± 4.30 VAS at night: 4.55 ± 2.75 CoS: 75.1 ± 81.85 SST: 77.49 ± 81.24	Overall population VAS at rest: 0.39 ± 0.77 VAS during movement: 1.87 ± 1.85 VAS at night: 0.7 ± 1.32 CoS: 84.07 ± 11.47 SST: 87.15 ± 14.99 Group I VAS at rest: 0.86 ± 0.99 VAS during movement: 1.77 ± 1.87 VAS at night: 0.91 ± 1.27 CoS: 75.10 ± 10.06 SST: 77.27 ± 16.7 Group II VAS at rest: 0.18 ± 0.56 VAS during movement: 1.89 ± 1.82 VAS at night: 0.59 ± 1.15 CoS: 85.37 ± 10.24 SST: 90.74 ± 13.34 Group III VAS at rest: 0.15 ± 0.49 VAS during movement: 1.9 ± 1.97 VAS at night: 0.65 ± 1.63 CoS: 92.45 ± 7.19 SST: 94.18 ± 7.69	No complications
Latini et al. (2024) [80]	21	2 and 12 weeks	Collagen injections	5 mg/1 mL of hydrolyzed bovine collagen	2 injections, at baseline and at 2 weeks	VAS: 63 ± 20.5 SPADI: 80.6 ± 21.5	VAS: 37 ± 23.3 SPADI: 50.3 ± 23.5	1 progression to FTRCT

RCTP = rotator cuff tendinopathy; PTRCT = partial-thickness rotator cuff tear; FTRCT = full-thickness rotator cuff tear; CoS = constant score; ASES = American shoulder and elbow surgeons score; VAS = visual analogue score; KSS = Korean shoulder scoring system; SST = simple *Shoulder* test; FVAS = function—VAS; PVAS = Pain—VAS; NRS = numerical rating scale; DASH = disability of the arm, shoulder, and hand; SPADI= shoulder pain and disability index.

**Table 2:** The results of the Modified Coleman Methodology Score (MCMS) used to assess the quality of the included articles.

Reference	Study Size	Follow-Up	N Procedures	Type of Study	Diagnostic Certainty	Description of Injection Technique	Rehabilitation and Compliance	Outcome Criteria	Outcome Assessment	Selection Process	Total
Kim et al., 2019 [12]	10	4	7	0	5	10	5	10	12	5	68
Kim et al., 2020 [79]	7	4	7	10	5	10	5	10	12	5	75
Chae et al., 2020 [48]	0	0	10	0	5	10	5	10	12	5	57
Corrado et al., 2020 [36]	0	0	10	0	5	5	5	10	12	5	52
Godek et al., 2022 [71]	7	0	7	15	5	5	5	10	12	5	71
Aldhafian et al., 2023 [77]	10	4	10	0	5	5	5	10	12	5	54
Buda et al., 2023 [49]	7	0	10	10	5	5	0	10	12	5	76
Latini et al., 2024 [80]	0	0	10	*10*	5	5	5	10	8	5	58
Maximum Score Possible	10	10	10	15	5	10	10	10	15	10	100
Mean ± Standard Deviation	5.1 ± 4.4	1.5 ± 2	8.8 ± 1.5	5.6 ± 6.2	5 ± 0	6.8 ± 2.6	4.3 ± 1.7	10 ± 0	11.5 ± 1.4	5 ± 0	63.8 ± 9.7

## Data Availability

The dataset is available upon request from the authors.

## Supplementary Materials

The following supporting information can be downloaded at: https://www.mdpi.com/article/10.3390/clinpract15020028/s1, Table S1: PRISMA checklist; Table S2: The Modified Coleman Methodology Score.
