# Supplementary material for: Collagen Injections for Rotator Cuff Diseases: A Systematic Review
Source: Clin Pract. 2025 Jan 28;15(2):28. doi: 10.3390/clinpract15020028 (PMC11854470; doi:10.3390/clinpract15020028)
Supplement: Supplementary file 1 [file clinpract-15-00028-s001.zip › clinpract-3377504-supplementary/Appendix B - MCMS.pdf]

Supplementary Material 2. The Modified Coleman Methodology Score

Part 1: One score for each of the sections:

1. Number of patients:	
a. <30	0
b. 30-50	4
c. 51-10	7
d. >100	10
2. Mean follow-up	
a. <12 months	0
b. 12-36 months	4
c. 37-60 months	7
d. >61 months	10
3. Surgical approach	
a. Different approaches and outcome not reported separately	0
b. Different approaches and outcome reported separately	7
c. Single approach	10
4. Type of study	
a. Retrospective cohort study	0
b. Prospective cohort study	10
c. Randomized controlled trial	15
5. Description of diagnosis	
a. Described without percentage specified	0
b. Described with percentage specified	5
6. Description of surgical technique	
a. Not stated/unclear – Inadequate	0
b. Only stated – Fair	5
c. Stated with details – Adequate	10
7. Description of postoperative rehabilitation	
a. Described	5
b. Not described	0

Part 2: Scores can be assigned for each option of every section

1. Outcome criteria	
a. Outcome measures clearly specified	2
b. Timing of outcome measures clear	2
c. Outcome measures with reported reliability	3
d. General health measure included	3
2. Outcome assessment	
a. Participants recruited	5
b. Investigator independent of surgeon	4
c. Written assessment	3
d. Assessment completed by patients	3
3. Description of participants selection process	
a. Selection criteria reported and unbiased	5
b. Recruitment rate reported (>90%)	5
c. Recruitment rate reported (<90%)	0

The figure reporting the Modified Coleman Methodology Score was retrieved from the following article: Mancino, F.; Di Matteo, V.; Mocini, F.; Cacciola, G.; Malerba, G.; Perisano, C.; De Martino, I. Survivorship and Clinical Outcomes of Proximal Femoral Replacement in Non-Neoplastic Primary and Revision Total Hip Arthroplasty: A Systematic Review. *BMC Musculoskeletal Disorders* **2021**, *22*, 933, doi:10.1186/s12891-021-04711-w.
